# Objective analysis of patient diaries used in an intensive care unit (ICU) in a London teaching hospital

**DOI:** 10.1186/2197-425X-3-S1-A719

**Published:** 2015-10-01

**Authors:** HT Tung, N Nordin, T Edginton, M Vizcaychipi

**Affiliations:** Imperial College, Faculty of Medicine, London, United Kingdom; Chelsea and Westminster Hospital NHS Foundation Trust, Department of Anaesthesia, London, United Kingdom; University of Westminster, Department of Psychology, London, United Kingdom; Chelsea and Westminster Hospital NHS Foundation Trust, London, United Kingdom

## Introduction

Patient diaries are used in the ICU setting to alleviate psychological distress in patients and their relatives.[1] Much work has been done to evaluate the effects of diaries, but little work has been done to examine protagonist involvement and the content of the narrative within the diaries.

## Objective

To formally analyse the structure of patient diaries and to develop a coding system to provide an objective method to evaluate patient experiences in ICU.

## Methods

31 patient diaries for admissions longer than 72 hours were included in the study. Patients with more than 1 ICU stay during their hospital admission were included independently. Diaries were handwritten by nurses and electronically transcribed, the analysis framework follows a previous ICU study documented in the literature.[2] Each diary was divided and coded into three phases, crisis phase (days 1-5), turning point phase (days 6-10) and normalisation phase (>10 days) and individual characteristics, overall structure and diary entries were coded and analysed independently.

## Results

Duration of admission ranged from 5 to 93 days. 26 of the 31 diaries that were analysed were started in the crisis phase. Analysis revealed that technical terms were used in 84% of diaries, with 45% of these entries observed in the normalisation phase. The majority of diaries (77%) had a summary of events within the crisis phase and only 1 diary (3%) failed to summarise events prior to admission. Notably 45% of diaries ended abruptly without any explanation to the patient, and surprisingly doctors were only rarely mentioned. Groups were also categorised based on the active role of the narrator. There was a significant difference between nurses, patients and families in all phases with 90% of nurses as active protagonists in the crisis phase, 33% active in the normalisation phase with only 2 patients being active protagonists in the normalisation phase.

## Conclusions

Nurses were found to have an active role throughout each phase in the patients´ stay in intensive care. Unexpectedly patients did not play a more active role during the normalisation phase and doctors were mentioned in few of the diary entries. Since diaries are meant for patients, it is important to consider that overuse of technical terms and the failure to include a summary of previous events and future plans after discharge from ICU may reduce their usefulness for patients.
